# Role of IL-17 Pathways in Immune Privilege: A RNA Deep Sequencing Analysis of the Mice Testis Exposure to Fluoride

**DOI:** 10.1038/srep32173

**Published:** 2016-08-30

**Authors:** Meijun Huo, Haijun Han, Zilong Sun, Zhaojing Lu, Xinglei Yao, Shaolin Wang, Jundong Wang

**Affiliations:** 1 Shanxi Key Lab of Environ-Veterinary Science, College of Animal Science and Technology, Shanxi Agricultural University, Taigu, Shanxi, 030801, People’s Republic of China; 2 Beijing Advanced Innovation Center for Food Nutrition and Human Health, College of Veterinary Medicine, China Agriculture University, Beijing, 100193, People’s Republic of China

## Abstract

We sequenced RNA transcripts from the testicles of healthy male mice, divided into a control group with distilled water and two experimental groups with 50 and 100 mg/l NaF in drinking water for 56 days. Bowtie/Tophat were used to align 50-bp paired-end reads into transcripts, Cufflinks to measure the relative abundance of each transcript and IPA to analyze RNA-Sequencing data. In the 100 mg/l NaF-treated group, four pathways related to IL-17, TGF-β and other cellular growth factor pathways were overexpressed. The mRNA expression of *IL-17RA*, *IL-17RC*, *MAP2K1*, *MAP2K2*, *MAP2K3* and *MAPKAPK2*, monitored by qRT-PCR, increased remarkably in the 100 mg/L NaF group and coincided with the result of RNA-Sequencing. Fluoride exposure could disrupt spermatogenesis and testicles in male mice by influencing many signaling pathways and genes, which work on the immune signal transduction and cellular metabolism. The high expression of the IL-17 signal pathway was a response to the invasion of the testicular immune system due to extracellular fluoride. The PI3-kinase/AKT, MAPKs and the cytokines in TGF-β family were contributed to control the IL-17 pathway activation and maintain the immune privilege and spermatogenesis. All the findings provided new ideas for further molecular researches of fluorosis on the reproduction and immune response mechanism.

Epidemiological investigations showed male infertility, low birth rates and abnormalities in sperm morphology, oligospermia and azoospermia occurring in areas with a high fluoride content^1^,^2^,^3^,^4^,^5^,^6^,^7^. Even in 2008 and 2009, Bruce Spittle, Managing Editor of the Journal Fluoride, published two articles in two consecutive years and emphatically stated the adverse effects of fluoride on male reproductive function^7^,^8^. Further studies in animals indicated a negative impact of fluoride ingestion on testicular histology, the structure of hypothalamus-pituitary-testicular and sperm quality^9^, including: sperm malformation^7^,^10^,^11^,^12^, sperm density, motility and activity^8^,^13^,^14^, spermchemotaxis^15^, sperm hyperactivation, capacitation, acrosome reaction and fertilizing ability^16^.

But how does fluoride affect male reproductive function with regard to the mechanism? It has been widely reported in the male reproductive systems that spermatogenesis was formed in testes and regulated by the hormone testosterone, cytokines and gene and protein expressions^17^,^18^. Meanwhile, our previous studies and many hazard identification studies also revealed that high doses of fluoride in animals affect potentially sensitive reproductive-tract targets and pathways, such as the reduction of antioxidant defenses, the enhancement of oxidative stress, and changes in the testicular cell cycle^19^,^20^,^21^,^22^. Overall, a number of studies have indicated fluoride exposure disrupts testicular development, but most of them focused on pathological observation and a limited number of genes so that up to now the specific molecular mechanisms of fluoride-induced spermatogenesis dysfunction were not clear.

Due to the complexity of molecular mechanisms, high throughput methods are quite necessary and playing more and more important roles in the research of toxicology. Moreover, the matured transcriptome sequencing (RNA-Seq) platforms have been successfully applied to the detection of gene fusions in cancer^23^, the analysis of vaccinia virus and host cell transcriptomes^24^ and the quantitative calculation of the abundance of expressed genes among the tissue transcriptome sequence data^25^. Thus, the deep RNA-Seq was applied to focus on genes and biological pathways affected by fluoride using and experimental validation to uncover the molecular basis of reproduction and sperm metabolism disorder. Delineating the overall gene expression profile in the testicles of experimental fluorosis mice will help to deeply identify the mechanisms involved in reproductive toxicity and other pathological disorders associated with fluoride.

## Methods

### Animals

Sixty adult male Kunming mice (aged 8 weeks, 25–26 g b.w.) were purchased from the Experimental Animal Center of Shanxi Medical University (Taiyuan, China), housed in standard plastic cages, maintained in a temperature-controlled environment (22–25 °C) with a 12 h light/dark cycle and fed a standard mouse diet and water ad libitum. After one-week acclimation, these mice were divided randomly into three groups of 20 each: a control group, which drank distilled water, and other two treatment groups, which received 50 and 100 mg/l NaF in their drinking water. The doses were chosen on the basis of the previous studies and considered the toxicities of fluoride in the male reproductive system^26^,^27^,^28^. To make sure of more than four spermatogenic cycles in mice (approximately 40 days)^13^, animals were treated with fluoride for 56 days. It’s important to note that in our previous research no significant differences were observed in body weight and major organ coefficient (testis/body weight) compared with the control group after 56 days fluoride exposure^9^,^15^. Based on that, we can probably rule out the influence of body weight change on the testicular in itself. Meanwhile, the formal study has found the decreased sperm and disorganized spermatogenic cells with the morphological observations of testis, more severely in high F group. And in Sertoli cells, we observed the density of cytoplasm decreased, heterochromatin gathered and mitochondria appeared with numerous vacuoles. All of these provided the support for sequence analysis.

Statement: All experimental procedures were conducted and performed as the policies for animal care and use encompass regulations approved by the Institutional Animal Care and Use Committee of Shanxi Agricultural University, including Animal Welfare Act, Guide for the Care and Use of Laboratory Animals and Guide for the Care and Use of Agricultural Animals in Research and Teaching.

### RNA Sample Preparation

All mice were killed by cervical dislocation on the 56th day, testicles were immediately isolated, frozen in liquid nitrogen and stored at −80 °C for RNA extraction and gene expression research. Total RNA was extracted from five tissue samples in each group using TRIzol (Invitrogen, Carlsbad, CA, USA) according to the protocol provided by the manufacturer. The quality and purity of total RNA were monitored by Nanodrop ND-2000 spectrophotometer (Nanodrop Technologies Inc., DE, USA) and electrophoresing on a 0.8% agarose gel (Sigma, St. Louis, MO), respectively.

### RNA Deep Sequencing

Based on the recently published papers, RNA deep sequencing was conducted as follows^29^,^30^. Briefly, the sequencing library of each RNA sample was prepared with the TruSeq RNA Sample Preparation Kit according to the manufacturer’s instructions (Illumina, San Diego, CA). The enriched libraries were diluted with elution buffer to a final concentration of 10 nM. Each sample (ca. 7 pM concentration) was subjected to 50 cycles of sequencing from both ends by the Illumina Hiseq^TM^ 2000 sequencing technology. Before doing any further analysis, FastQC (http://www.bioinformatics.bbsrc.ac.uk/projects/fastqc/) was applied to perform quality control checks on raw sequence data coming from high throughput sequencing pipelines.

Following deep sequencing analysis of 50-bp length paired-end reads, Bowtie and Tophat were used to align the reads into transcripts based on the Mouse Reference Genome (ftp://ftp.cbcb.umd.edu/pub/data/bowtie_indexes/m_musculus_ncbi37_c.ebwt.zip). To measure the relative abundance of each transcript, the resulting aligned reads were analyzed with Cufflinks suite (http://cufflinks.cbcb.umd.edu). Expression of each transcript was quantified as the number of reads mapping to a gene divided by the gene length in kilobases and the total number of mapped reads in millions, and designated as fragments per kilobase of exon per million fragments mapped (FPKM).

### Gene Annotation and Expression Profiling Analysis

The Ensembl Transcript ID was used as the primary identifier for all analyses. When multiple splice variants existed, all of them were selected. In generating the FPKM distributions of intergenic regions, regions with a distance of at least 10 kb from any RefSeqor Ensembl gene were selected. The annotation information corresponding to each Ensembl Transcript ID was retrieved from the Ensembl database via BioMart (http://www.biomart.org/biomart/martview).

For each fluoride concentration of interest, all the transcripts were pulled from the file generated by Cufflinks. The measurements with RPKM values close to zero (approximately 5% of the total) were discarded. The RPKM values were logarithmically transformed to base 2, and the measurements of each transcript within an experimental group were subjected to outlier detection^29^,^30^.

### Enriched Biochemical Pathways in the Fluorosis Testicle

Ingenuity Pathway Analysis (IPA, Ingenuity System Inc, USA, http://www.ingenuity.com/) were used to alter the significant genes, identify global canonical pathways and dynamically generate biological networks in the testicle of experimental fluorosis mice^31^,^32^. The core component of IPA is the Ingenuity Pathways Knowledge Base (IPKB), which contains the biological function, interaction, and related information of a curated gene set and more than 330 biochemical pathways^31^. Using the whole gene set of IPKB as the background, the genes with their symbols and the corresponding GenBank accession numbers were uploaded into the IPA with a view to revealing the enriched biochemical pathways^29^,^30^. All the pathways with one or more genes overlapping the candidate genes were extracted. In IPA, each of the canonical pathway was assigned a *P* value via Fisher’s exact test, which denoted the probability of overlap between the pathway and input genes^33^. Reported significance was defined as *P* < 0.05 with a fold change (FC) larger than 1.5.

### Quantitative Real Time RT-PCR

Quantitative real-time RT-PCR (qRT-PCR), the traditional quantification method on gene expression, was adopted to further confirm the findings from the RNA-seq analysis. On the basis of the results, we optionally detected several significant genes involved in the IL-17 signaling pathway with the same RNA samples used for RNA-seq analysis, considering they are the only pathways showing differences in the testicle of all the fluorosis groups. These genes and their primers used in the qRT-PCR array were listed in Supplementary Table S1. Primers were designed using Primer Express v. 3.0 software (Applied Biosystem Inc., CA, USA). The qRT-PCR analysis was conducted in a total volume of 10 μl containing 5 μl 2 × SYBR Premix Ex Taq^TM^ (Takara Bio Inc., China), combined with sense and antisense primers (0.4 μl, final concentration 250 nM), and 1 μl diluted cDNA in a 384-well plate using the Applied Biosystems QuantStudio^TM^ 7 Flex Real-Time PCR System (Thermo Fisher Scientific, USA). The conditions for real-time PCR were as follows: after initial denaturation at 95 °C for 15 s, 55 PCR cycles were started with thermo cycling conditions at 95 °C for 5 s, 61 °C for 15 s, and 72 °C for 6 s, and then a melting curve analysis was performed to verify the specificity of the PCR product. Every sample was analyzed in triplicate. System software and the 2^−ΔΔCt^ method were applied to quantitative calculation.

### Data Analysis for qRT-PCR

All data were conducted by GraphPad Prism 5 software (GraphPad Software Inc., San Diego, USA). Statistical analysis was performed by one-way ANOVA and followed by a Tukey’s test. All values in the experiment were expressed as mean ± SEM (standard error of the mean) and values of *P* < 0.05 were expressed statistically significant (n = 5 per group).

## Result

### Overview of Sequencing Data from RNA-seq Analysis

Sequencing and mapping quality were analyzed using FASTQC. Total sequences among samples ranged from 30 to 37 million reads, with an average of approximately 31 million raw reads per sample. About 23–32 × 10^6^ reads (82–84% of the total raw reads) were uniquely aligned to mouse genome sequence among samples, with an average of 24 × 10^6^ reads per sample.

### Identification of Genes and Pathways Altered in Testicle

**Figure 1:**
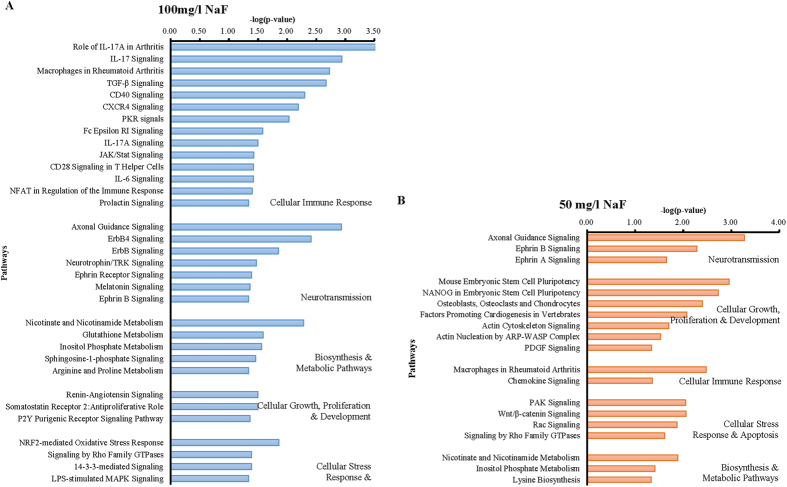
Enriched biochemical pathways altered in the testicle in the 50 mg/l and 100 mg/l NaF-treated groups compared to the control groups.

Analysis of the data indicated that there were 120 and 298 differentially expressed genes in the 50 mg/l and 100 mg/l NaF-treated groups, respectively. To further understand these changes at the pathway level, an IPA analysis was conducted and showed 99 and 246 genes mapped with corresponding GenBank, meanwhile, 19 and 33 signaling pathways to be significantly altered in the two treated groups separately (Fig. 1).

In the overview, a huge variety of the pathways with the different biological functions were identified by IPA in each treatment group. Study found that in the 50 mg/l NaF-treated group there was a significant influence on the communication signals between cells and cell biological mechanisms, related to the cellular growth, proliferation & development. However, it was noteworthy that 14 signaling pathways mainly focused on the immune responses in the 100 mg/l NaF-treated group, and among them the interleukin-17 (IL-17), IL-17A and other two signaling transduction pathways presented the close association with the regulation of the IL-17 family and IL-17 receptors, which laid the foundation for validating the predicted genes.

**Figure 2:**
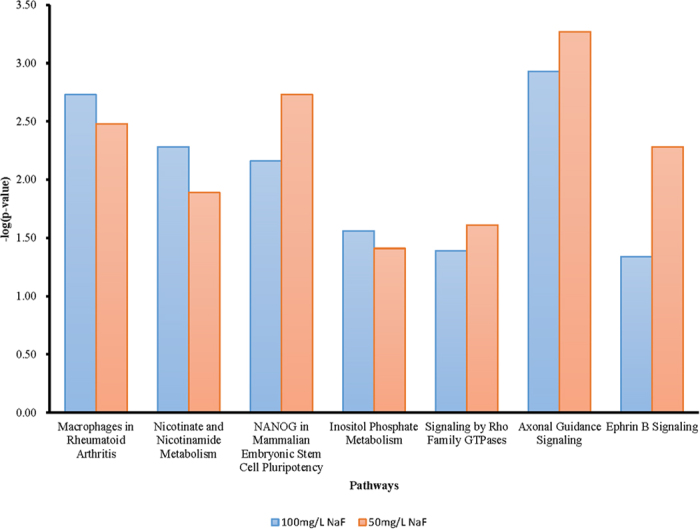
The common significant pathways between the 50 mg/l and 100 mg/l NaF-treated groups.

There were 7 signaling pathways altered in both the NaF-treated groups as listed in Fig. 2. These signals were highly related with oxidative stress, cell development and cell apoptosis. Axonal Guidance Signaling and EphrinB Signaling pathway were associated with neurotransmission and altered in both the treatments.

**Figure 3:**
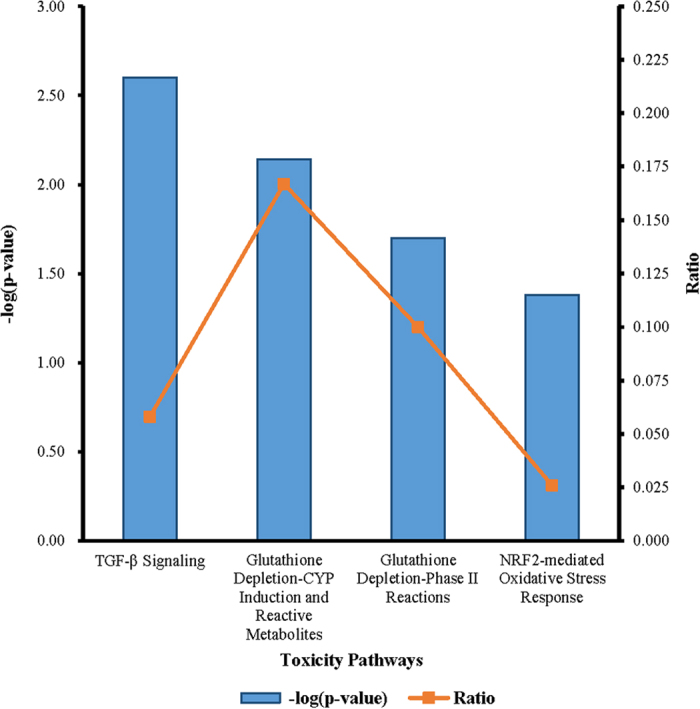
The significant pathways related to toxicology altered in the 100 mg/l NaF-treated group.

In addition, for further information about the relationships between the differentially expressed genes and toxicology, 36 signaling pathways interacted in toxicity and were identified by IPA in the 100 mg/l NaF-treated group. Among them, four pathways were significantly altered, as listed in Fig. 3. Also four of them were highly relevant to cell differentiation and apoptosis as well as oxidative metabolism, including the transforming growth factor beta (TGF-β) signaling pathway, glutathione depletion reactions and NRF2-mediated oxidative stress response, especially the TGF-β signaling pathway involved in many cellular processes and commonly inducing the production of cytokines.

### Description of Important and Representative Genes in Testicle

**Figure 4:**
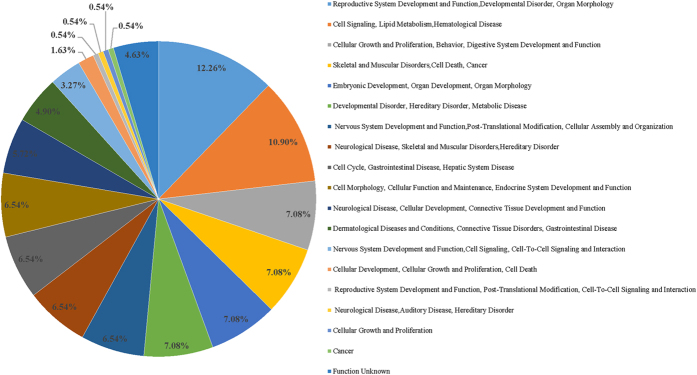
The percentage of different functional genes in all the 367 DEGs screened in the 50 mg/l and 100 mg/l NaF-treated groups. It not only presented the relationship among genes and functions, diseases, but also describes the important role of genes on the development the reproductive system, nervous system, skeletal and muscular disorders, cell signaling and other metabolic processes.

In the perspective of gene functions, based on gene function annotation by IPA, 367 differentially expressed genes (DEGs) were screened both in the 50 mg/l and 100 mg/l NaF-treated groups when compared with the control group. All the genes were grouped into various functional categories, and the relationship among genes and functions, diseases were presented accurately. In Fig. 4, the percentage of genes that worked on the reproductive system development and function reached up to 12.26%. About 10.90% and 7.08% were related to cell signaling and cellular growth and proliferation, respectively. More than 7.08% genes worked on skeletal and muscular disorders. Almost 56.38% genes were participating in the process of cell metabolism. It was found that the same gene may plays a different role in multiple metabolic processes.

**Figure 5:**
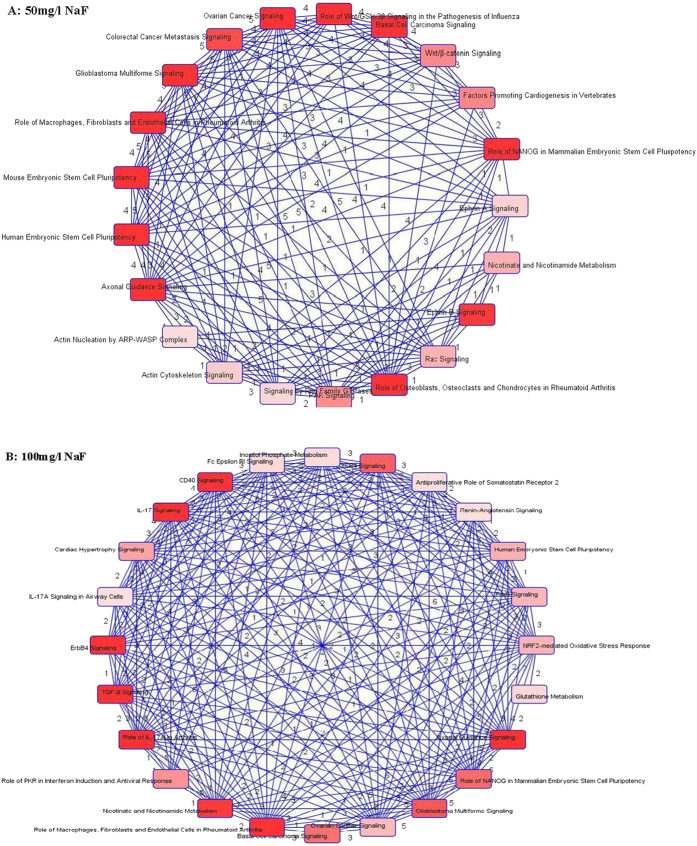
Detected interactions of enriched pathways in the testicle in the 50 mg/l and 100 mg/l NaF-treated groups. All significant pathways identified by IPA software were highly interrelated. The number represented the count of the common genes between two pathways.

**Figure 6:**
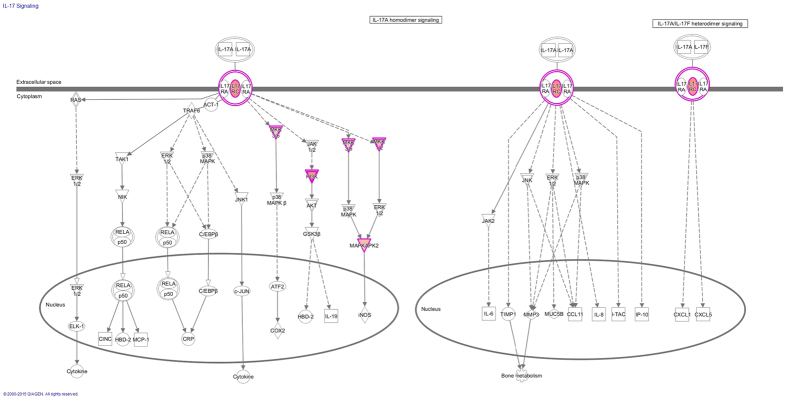
The map of the intracellular metabolic processes for IL-17 signaling pathway. The genes marked with red color indicated their value of genetic expression were significantly changed in the 100 mg/l NaF group. MAP2K3/MAP2K6marked by MKK3/6, MAP2K1/MAP2K2 marked by MKK1/2, and PIK3R1 marked by PI3K.

In the perspective of pathways, shown in the Fig. 5, it has already suggested a correlation among the high-expression pathways identified by IPA software. These figures told us that many common genes were working on the regulation by the different signal pathways. Therefore, the interaction of multiple genes were concerned. Just as shown in the Table 1, in the testicle of mice treated with different fluoride concentrations, there were nine important and representative genes that appeared in more than four pathways, even MAP2K2 were directly involved in the regulation of almost 20 significant pathways. All of MAP2K2, PIK3R1, MAP2K3, MAPKAPK2 and IL17RC participated in the IL-17 intracellular metabolic processes (Fig. 6).

### Expression Analysis by qRT-PCR

**Figure 7:**
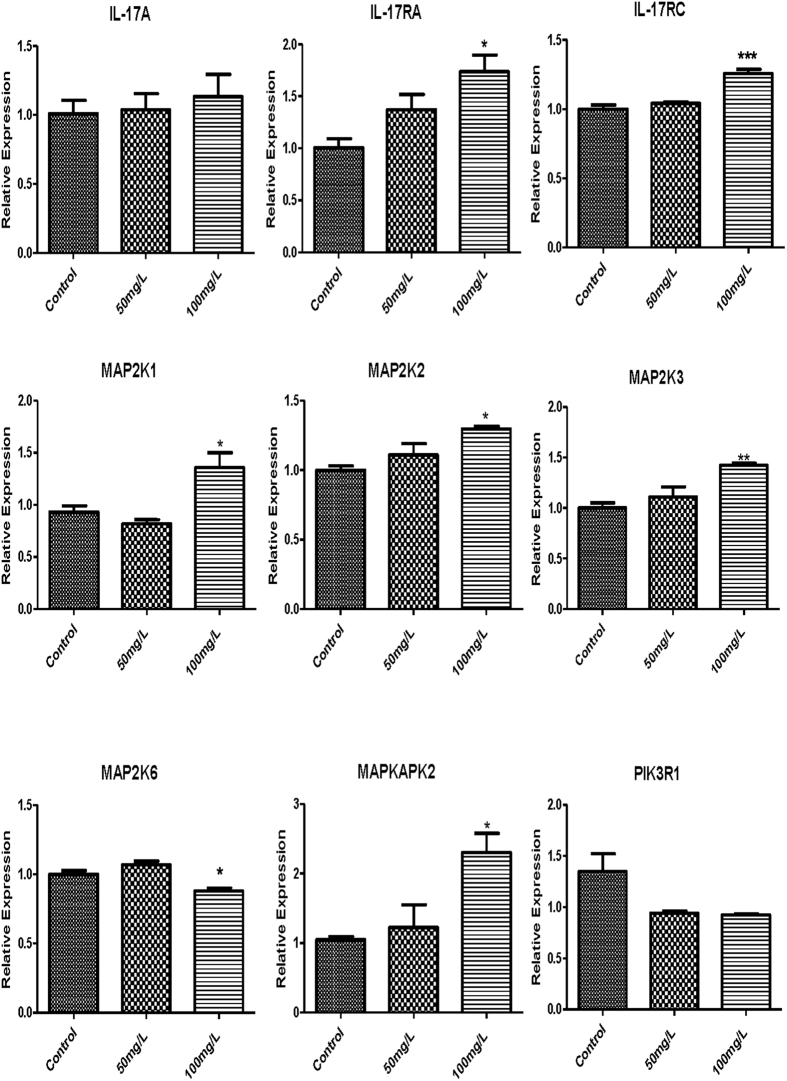
Results of the nine mRNA expression related to the IL-17 signaling pathway in testis of mice in each treatment group. Compared with the control group, *P < 0.05, **P < 0.01, ***P < 0.001, n = 5.

Based on the RNA-seq analysis of the pathways, nine genes, including *IL17A*, *IL17RA*, *IL17RC*, *MAP2K3*, *MAP2K6*, *PIK3R1*, *MAPKAPK2*, *MAP2K1* and *MAP2K2*, representing the IL-17 signaling pathway described above were selected for confirmation as well as to monitor their expression with qRT-PCR, and the data was statistically analyzed as follows (Fig. 7).

Compared with control group, in the 100 mg/l NaF-treated group the mRNA expression level of *IL17RA*, *IL17RC*, *MAP2K1*, *MAP2K2*, *MAP2K3* and *MAPKAPK2* increased remarkably. The gene expression of *MAP2K6* and *PIK3R1* reduced gradually. There was not significant changes in the gene expression of 50 mg/l NaF-treated group.

Besides that, the linear regression analysis of the fold change of the gene expression ratios between RNA-seq and qRT-PCR showed significantly positive correlation (Supplementary Fig. S1), confirming our transcriptome analysis.

## Discussion

Although fluoride is safe and even healthy at low concentrations, sustained consumption of large amounts of soluble fluoride salts is dangerous. It was well known that toxic levels of fluoride exposure over a long period of time can adversely cause skeletal and tooth fluorosis induced by oxidative stress of osteoblasts and osteoclasts^34^,^35^,^36^. It also can lead to some adverse effects on a number of physiological functions, for example, thyroid dysfunction^37^, nephrotoxicity^35^,^38^, cardiometabolic risk^39^,^40^, neurodevelopmental disorder in juvenile stage^38^,^41^,^42^ and even male reproductive endocrine disruption^7^,^8^.

However, the mechanisms of reproduction injury induced by taking in excess fluoride were still inconclusive. Attempting to address the root cause, this experiment was the first time using the transcriptome sequencing in the testicle of experimental fluorosis mice to explore the relative gene expression levels in mouse testis and interpret the effect of fluoride poisoning in the male reproductive system. Different from earlier studies, our study considered the damages of fluoride on the male reproductive system holistically, including a variety of pathways and genes, rather than just a single factor.

Generally, the testis and the capacity of sperm were of the important indices for evaluating the reproductive system. The testis comprises mostly seminiferous tubules and interstitial cells, localized between seminiferous tubules, to produce and secrete testosterone^43^. The epithelium of the tubule consists of a type of sustentacular cells known as Sertoli cells, which differentiate through meiosis into sperm cells. During spermatogenesis, the main function of Sertoli cells is to nourish the developing sperm cells and also act as phagocytes, consuming the residual cytoplasm and secreting the inhibin, activins and androgen binding protein^44^. While our previous studies reported that the pathologic and morphological changes of chronic fluorosis in testicles and sperm were observed. The cavitation of seminiferous tubules, cellular atrophy and other structural damages can result in the reduction of androgen binding protein synthesis and the inadequate amounts of testosterone, which, in turn, can cause spermatogenesis to be blocked and spermatid developed abnormally with different morphology. Song Ke qin *et al*.^45^ also found the distention and vesiculization of smooth endoplasmic reticulum and the deposition of large lipid droplets appearing in the Sertoli cells under the ultrastructural observations of rat testes. So what happened in these cells?

Mendoza-Schulz A. *et al*.^46^ said that fluoride had significantly effect on hormone secretion and protein synthesis in the endocrine cells. They found the changes in phosphorylation status of both cytoskeletal and cytosolic protein fractions, as well as in actin cytoskeletal arrangements were observed. Similarly, in our research of the 50 mg/l NaF-treated group, actin nucleation and actin cytoskeleton signal pathways were stimulated with the significant expression of growth factors. In response to these microenvironmental and functional alterations, immune cells often represent dramatically change their functional activities to reprogram their cellular metabolism and release the metabolic stresses^47^. A lymphocyte, such as T cells, transforms from a relatively inert cell to a cell engaging in robust growth and proliferation, often producing large amounts of effector molecules, including cytokines^47^. Yet despite all that, there is emerging evidence that metabolic enzymes and regulators can also have a direct role in controlling immune cell functions^47^. For instance, in CD4 T cells, GAPDH has been described to bind to IL2 mRNA and inhibit translation. Accordingly, the testicular immunological efficiency was enhanced along with the increase of doses of fluoride.

Although the testis is an immune privileged organ and the most important spaces of spermatogenesis and steroidgenesis, toxic agents and inflammation may overwhelm immune suppressor mechanisms inducing autoimmune reactions against spermatic antigens which result in aspermatogenesis and infertility^18^. The cytokine interleukin-17, ukin-17 (IL-17 or IL-17A) and the pathology associated with aberrant IL-17 signaling played an important role in maintaining the testicular immune including cell immunity, mucosal immunity and cytokines, especially in experimental autoimmune orchitis (EAO)^18^,^48^,^49^. Jacobo P. *et al*.^49^ reported that in EAO testis developed by active immunization with spermatic antigens, testis-infiltrating cells revealed an increased number of macrophages, dendritic cells and T cell subsets including Th17 cells so that TNF-α, IL-17 and other immune cells secreted pro-inflammatory cytokines, which disrupted the normal testicular immune suppressor microenvironment. And they said IL-17 cells in EAO testis have a mature immunogenic status and are able to induce immune responses to testicular antigens.

In many cases, an excess of IL-17 is associated with abnormal inflammation, implicated in rheumatoid arthritis, asthma, psoriatic arthritis, ankylosing spondylitis, systemic lupus erythematosus and autoimmune encephalomyelopathy, which, not surprisingly, have become a major therapeutic target for these diseases. It have been recently found Th17 cells are a subset of T helper cells and play important functions in host defense and the pathogenesis of various human autoimmune and inflammatory diseases^50^,^51^,^52^. Th 17 cells could produce IL-17A, who would mediate many of the downstream pathologic functions in the cells. IL-17A utilizes IL-17RA and IL-17RC as its receptors that are mainly expressed on tissue epithelial cells and fibroblasts. While IL-17A is important for host defense against many extracellular pathogens, they can also cause excessive tissue damage and exacerbate proinflammatory responses during autoimmunity^52^. Therefore, as for our study, the IL-17 signal pathway and its proinflammatory cytokines were expressed in higher levels in high fluoride-exposed testis (100 mg/l NaF). It was the response to the invasion of the immune system by extracellular fluoride and involve in the maintenance of testicular immune privilege and spermatogenesis^53^. And the activation of toll-like receptors IL-17RA and IL-17RC suggested that these cells played important roles in protecting the seminiferous epithelium from invading fluoride.

During this study we also found the transforming growth factor beta (TGF-β) signaling pathway has the most important research value in the toxicology field. TGF-β signaling pathway is involved in many cellular processes in both the adult organism and the developing embryo including cell growth, cell differentiation, apoptosis, cellular homeostasis and other cellular functions^54^,^55^,^56^. TGF-βs belong to a family of the immunosuppressive and anti-inflammatory TGF superfamily and widely distributed in embryonic and adult tissues^18^. Most TGF-βs are present in the testis as the latent inactive precursor form and are expressed constitutively at high levels being produced mainly by Sertoli cells, Leydig cells, and peritubular, but in post pubertal testis, cytokines of the TGF-β family are also expressed by early spermatids and spermatocytes. Once activated at its site of action by local proteases, TGF-βs would contribute to the immunological privileged site of the testis through their strong immunosuppressive ability^18^,^52^. Thus, what we think is that the gene activation of TGF-β family have help to sustain the immune exemption of testicle in fluorosis.

In addition, based on the expression of *MKK3/6*, *MKK1/2* and *PI3K* in RNA-seq and the intracellular metabolic processes of IL-17 signaling pathway, we could infer that the IL-17 family members took part in the activation of the Mitogen-activated protein (MAP) kinase pathway and PI3 Kinase-AKT pathway, which are involved in the regulation of a variety of growth and differentiation pathways through several phosphorylation cascades^52^,^57^. The MAP signaling cascade is activated by a number of receptors: the extracellular mitogen binds to the membrane receptor, then this allows Ras (a GTPase) to swap its GDP for a GTP, and activate MAP3K, which activates MAP2K, which activates MAPK, finally MAPK can activate a transcription factor^58^,^59^. MAPK-ERK1/2 played an important role in the regulation of cell growth and cell cycle progression. PI3-kinase and its downstream kinase AKT are potent inhibitors of apoptosis in many cell types. AKT is phosphorylated upon IL-17stimulation and also adds to the possible involvement of PI3-kinase in the propagation of signal through the IL-17R^52^. Together, these results indicated that PI3-kinase/AKT and MAPKs serves as the upstream arbitrator of the IL-17 pathway activation and had contributed to the increased binding of the inflammatory transcription factor in IL-17 pathways.

Anyway, all the found helped us to better understand the molecular basis of reproduction and sperm metabolism disorder and deeply identify the mechanisms involved in reproductive toxicity and other pathological disorders associated with fluoride. At first, the aim we were pursuing was to find really reliable molecules and genes associated with reproduction by RNA direct sequencing of testis. However, what we got was quite surprising: a plenty of other metabolic pathways and classic genes of the systemic and comprehensive responses were dig out. Peeping a spot to see overall picture: local delicate change was packed with the complex issues of the whole organism. But for the further verification and exploration, researches on the cellular level and the significant expression of proteins during the spermatogenesis should be carried out.

## Conclusions

The high expression of genes in the IL-17 signal pathway was the response to the invasion of the testicular immune system by extracellular fluoride. The cytokines of the TGF-β family performed key roles in the maintenance of immune privilege and spermatogenesis. Meanwhile, PI3-kinase/AKT and MAPKs acted as the upstream arbitrator of the IL-17 pathway activation and have contributed to the increased binding of the inflammatory transcription factor in IL-17 pathways.

All the findings, including the metabolic pathways and classic genes, could provide new ideas and clues for further researches of the molecular mechanism of fluorosis on the field of reproduction and development, immune response, oxidative stress, cell regulation mechanism and so on.

## Additional Information

**How to cite this article**: Huo, M. *et al*. Role of IL-17 Pathways in Immune Privilege: A RNA Deep Sequencing Analysis of the Mice Testis Exposure to Fluoride. *Sci. Rep*. **6**, 32173; doi: 10.1038/srep32173 (2016).

## Supplementary Material

Supplementary Information

## Figures and Tables

**Table 1:** Important and representative genes altered in the fluorosis testicle based on the significant pathways of 100 mg/l NaF-treated group.

#	Symbol	Matched Pathway	Entrez Gene Name	Ensembl	Fold Change	p-value	Location	Type(s)
1	MAP2K2(20)	Role of IL-17A in Arthritis	mitogen-activated protein kinase kinase 2	ENSMUST00000105331	2.038	4.49E-02	Cytoplasm	kinase
		IL-17 Signaling						
		Axonal Guidance Signaling						
		Role of Macrophages, Fibroblasts and Endothelial Cells in Rheumatoid Arthritis						
		TGF-β Signaling						
		ErbB4 Signaling						
		CD40 Signaling						
		Nicotinate and Nicotinamide Metabolism						
		Glioblastoma Multiforme Signaling						
		CXCR4 Signaling						
		Role of NANOG in Mammalian Embryonic Stem Cell Pluripotency						
		Cardiac Hypertrophy Signaling						
		NRF2-mediated Oxidative Stress Response						
		ErbB Signaling						
		Ovarian Cancer Signaling						
		Fc Epsilon RI Signaling						
		Inositol Phosphate Metabolism						
		Antiproliferative Role of Somatostatin Receptor 3						
		IL-17A Signaling						
		Renin-Angiotensin Signaling						
2	PIK3R1(16)	Role of IL-17A in Arthritis	phosphoinositide-3-kinase, regulatory subunit 6	ENSMUST00000060441	2.935	5.84E-03	Cytoplasm	kinase
		IL-17 Signaling						
		Axonal Guidance Signaling						
		Role of Macrophages, Fibroblasts and Endothelial Cells in Rheumatoid Arthritis						
		ErbB4 Signaling						
		CD40 Signaling						
		Glioblastoma Multiforme Signaling						
		CXCR4 Signaling						
		Role of NANOG in Mammalian Embryonic Stem Cell Pluripotency						
		NRF2-mediated Oxidative Stress Response						
		ErbB Signaling						
		Fc Epsilon RI Signaling						
		Inositol Phosphate Metabolism						
		Antiproliferative Role of Somatostatin Receptor 4						
		IL-17A Signaling						
		Renin-Angiotensin Signaling						
3	MAP2K3(11)	Role of IL-17A in Arthritis	mitogen-activated protein kinase kinase 3	ENSMUST00000019076	1.658	4.12E-02	Cytoplasm	kinase
		IL-17 Signaling						
		Role of Macrophages, Fibroblasts and Endothelial Cells in Rheumatoid Arthritis						
		TGF-β Signaling						
		CD40 Signaling						
		Nicotinate and Nicotinamide Metabolism						
		Role of PKR in Interferon Induction and Antiviral Response						
		NRF2-mediated Oxidative Stress Response						
		ErbB Signaling						
		Fc Epsilon RI Signaling						
		Inositol Phosphate Metabolism						
4	WNT6(5)	Axonal Guidance Signaling	wingless-type MMTV integration site family, member 6	ENSMUST00000006716	2.945	4.93E-04	Extracellular Space	other
		Role of Macrophages, Fibroblasts and Endothelial Cells in Rheumatoid Arthritis						
		Glioblastoma Multiforme Signaling						
		Role of NANOG in Mammalian Embryonic Stem Cell Pluripotency						
		Human Embryonic Stem Cell Pluripotency						
5	WNT9A(4)	Axonal Guidance Signaling	—	ENSMUST00000108783	2.205	3.52E-02	Other	other
		Role of Macrophages, Fibroblasts and Endothelial Cells in Rheumatoid Arthritis						
		Glioblastoma Multiforme Signaling						
		Role of NANOG in Mammalian Embryonic Stem Cell Pluripotency						
6	WNT10A(4)	Axonal Guidance Signaling	wingless-type MMTV integration site family, member 10A	ENSMUST00000006718	1.973	2.21E-02	Extracellular Space	other
		Role of Macrophages, Fibroblasts and Endothelial Cells in Rheumatoid Arthritis						
		Glioblastoma Multiforme Signaling						
		Role of NANOG in Mammalian Embryonic Stem Cell Pluripotency						
7	GNA11(4)	Axonal Guidance Signaling	guanine nucleotide binding protein (G protein), alpha 11 (Gq class)	ENSMUST00000043604	2.433	9.84E-03	Plasma Membrane	enzyme
		Role of Macrophages, Fibroblasts and Endothelial Cells in Rheumatoid Arthritis						
		CXCR4 Signaling						
		Inositol Phosphate Metabolism						
8	MAPKAPK2 (4)	Role of IL-17A in Arthritis	mitogen-activated protein kinase-activated protein kinase 2	ENSMUST00000016672	1.991	7.62E-03	Nucleus	kinase
		IL-17 Signaling						
		Role of Macrophages, Fibroblasts and Endothelial Cells in Rheumatoid Arthritis						
		CD40 Signaling						
9	IL17RC(4)	Role of IL-17A in Arthritis	interleukin 17 receptor C	ENSMUST00000113062	3.053	1.21E-03	Plasma Membrane	transmembrane receptor
		IL-17 Signaling						
		Role of Macrophages, Fibroblasts and Endothelial Cells in Rheumatoid Arthritis						
		IL-17A Signaling						
