# Supplementary material for: Role of IL-17 Pathways in Immune Privilege: A RNA Deep Sequencing Analysis of the Mice Testis Exposure to Fluoride
Source: Sci Rep. 2016 Aug 30;6:32173. doi: 10.1038/srep32173 (PMC5004130; doi:10.1038/srep32173)
Supplement: Supplementary Information [file srep32173-s1.pdf]

Supplementary Information

Role of IL-17 Pathways in Immune Privilege: A RNA Deep Sequencing Analysis of the Mice Testis Exposure to Fluoride

Meijun Huo, Haijun Han, Zilong Sun, Zhaojing Lu, Xinglei Yao, Shaolin Wang, and Jundong Wang

Supplementary Table S1. Genes selected for QRT-PCR and Their Primer Sequences[a]

Symbol	Gene Name	NCBI Reference Sequence	Sequence (5'->3')	Length (bp)
β-actin			F: TACCACATCCAAGAAGGCAG	141
			R: TGCCCTCCAATGGATCCTC	
IL17A	IL-17A	NM_010552.3	F:AGCGTGTCCAAACACTGAGG	125
			R:ACGTGGAACGGTTGAGGTAG	
IL17RA	IL-17RA	NM_008359.2	F:ACCCAAACCACAAATCCAAG	128
			R:TGTGTCCAAGGTCTCCACAG	
IL17RC	IL-17RC	NM_134159.4	F:CCACAAGATTTCCAGTTGGTG	80
			R:GCTTGCAGCTGAACCTTCTC	
MAP2K3	MAP2K3	NM_008928.4	F:CCAGTTCTCCCCTGAGTTTG	111
			R:TTGTGCAAGGTGAAGAATGG	
MAP2K6	MAP2K6	NM_011943.2	F:AGTGGAGGCTGATGACTTGG	148
			R:CAGACGTTTCTGCTCCTGTG	
PIK3R1	P13K	NM_001024955.2	F:GGCAGAAGAAGCTGAACGAG	147
			R:GCAATAGGTTCTCCGCTTTG	
MAPKAPK2	MAPKAPK2	NM_008551.1	F:AACGATGGGAGGATGTCAAG	116
			R:CTTGAGAAGCAGAGGGTTGG	
MAP2K1	MKK1	NM_008927.3	F:ATCTTCGGGAGAAGCACAAG	135
			R:CGAAGGAGTTGGCCATAGAG	
MAP2K2	MKK2	NM_023138.4	F:ACCGGCACTCACTATCAACC	117
			R:TCATCCAGGTCCAGCTCTTC	

[a]*All the real-time PCR primers were provided by Life Technologies Corp. (Shanghai, China).*

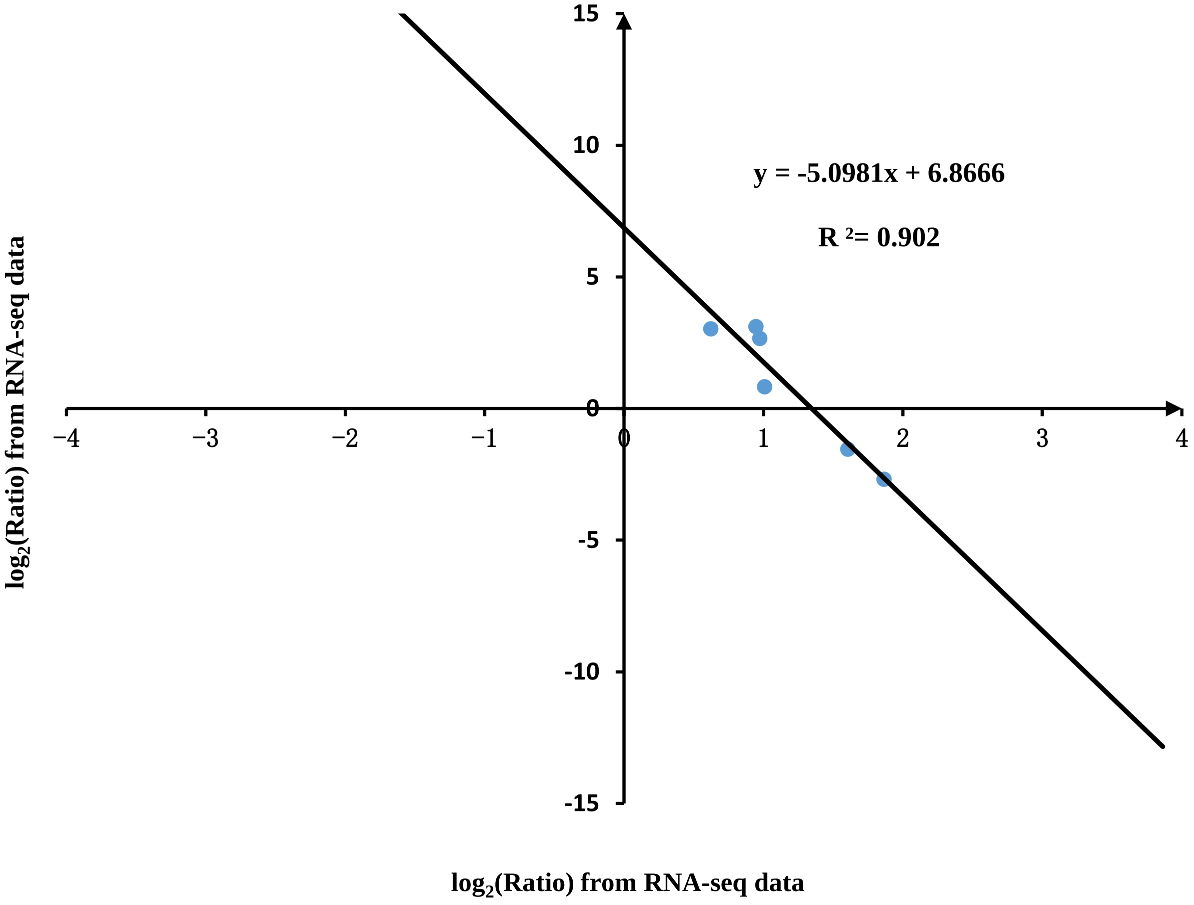

Supplementary Figure S1. Coefficient analysis of fold change data between qRT-PCR and RNA-seq. Data indicating relative transcript level from qRT-PCR are means of five replicates, and RPKMs from RNA-seq are means of three replicates. Scatterplots were generated by the log2 expression ratios from RNA-seq (x-axis) and qRT-PCR (y-axis).
